# The pharmacists’ interventions after a Drug and Therapeutics Committee (DTC) establishment during the COVID-19 pandemic

**DOI:** 10.1080/20523211.2024.2372040

**Published:** 2024-07-12

**Authors:** Amira B. Kassem, Ahmad Z. Al Meslamani, Dina H. Elmaghraby, Yosr Magdy, Mohamed AbdElrahman, Ahmed M.E. Hamdan, Hebatallah Ahmed Mohamed Moustafa

**Affiliations:** a Department of Clinical Pharmacy and Pharmacy Practice, Faculty of Pharmacy, Damanhour University, Damanhour, Egypt; b College of Pharmacy, Al Ain University, Abu Dhabi, United Arab Emirates; c AAU Health and Biomedical Research Center, Al Ain University, Abu Dhabi, United Arab Emirates; d Kafr El Dawar General Hospital, Department of infectious disease, Ministry of Health, Beheira, Egypt; e Clinical Pharmacy Department, College of Pharmacy, Al-Mustaqbal University, Babylon, Iraq; f Clinical pharmacy Department, Badr University Hospital, Faculty of Medicine, Helwan University, Helwan, Egypt; g Department of Pharmacy Practice, Faculty of Pharmacy, University of Tabuk, Tabuk, Saudi Arabia; h Clinical Pharmacy and Pharmacy Practice Department, Faculty of Pharmacy, Badr University in Cairo, Badr, Egypt

**Keywords:** Antibiotics abuse, clinical pharmacists, COVID-19, Drug and Therapeutics Committee, intensive care unit, medication errors

## Abstract

**Introduction:**

Healthcare systems in developing countries faced significant challenges during COVID-19, grappling with limited resources and staffing shortages. Assessment of the impact of pharmaceutical care expertise, particularly in critical care units during the pandemics, in developing countries remains poorly explored. The principal aim of our study was to assess the impact of the Drug and Therapeutics Committee (DTC), comprising clinical pharmacists, on the incidence, types, and severity of medication errors and associated costs in using COVID-19 medications, especially antibiotics.

**Methods:**

An interventional pre–post study was carried out at a public isolation hospital in Egypt over 6 months.

**Results:**

Out of 499 medication orders, 238 (47.7%) had medication errors, averaging 2.38 errors per patient. The most frequent were prescribing errors (44.9%), specifically incorrect drug choice (57.9%), excessive dosage (29.9%), treatment duplication (4.5%), inadequate dosage (4.5%), and overlooked indications (3.6%). Linezolid and Remdesivir were the most common medications associated with prescribing errors. Pharmacists intervened 315 times, primarily discontinuing medications, reducing doses, introducing new medications, and increasing doses. These actions led to statistically significant cost reductions (*p* < 0.05) and better clinical outcomes; improved oxygen saturation, decreased fever, stabilised respiratory rates, and normalised white blood cell counts. So, clinical pharmacist interventions made a notable clinical and economic difference (66.34% reduction of the expenses) in antibiotics usage specifically and other medications used in COVID-19 management during the pandemic.

**Conclusion:**

Crucially, educational initiatives targeting clinical pharmacists can foster judicious prescribing habits.

## 1. Introduction

The coronavirus disease 2019 (COVID-19) pandemic has caused a dramatic humanistic and economic burden globally (Alenzi et al., 2022; Lui et al., 2021; Peramo-Álvarez et al., 2021; Smith Jervelund & Eikemo, 2021). It posed unprecedented challenges to clinicians and healthcare systems aiming for optimal disease management, especially in low to middle-income countries (LMICs) and low-resource settings due to insufficient staffing, poor communication, and inadequate resources (Al Meslamani et al., 2021; Elhadi et al., 2020; Kajal et al., 2020; Kassem et al., 2021). Critically ill patients usually receive more medications than patients in other units and most of these medications are high-risk and/or intravenous, with frequent changes in medication regimens (Fair et al., 2023; Wang et al., 2020). Those critically ill patients are more vulnerable to medication errors, which may result in serious adverse events, threaten their safety, and jeopardise the quality of care they receive (Di Simone et al., 2016; Suclupe et al., 2020).

Amid the COVID-19 pandemic, another silent pandemic, that of antibiotic resistance due to antibiotic overuse and misuse, was threatening the globe (Elsayed et al., 2021; Founou et al., 2021; Garg, 2021). Egypt is in the middle of the list of many countries in terms of antibiotic consumption expressed in defined daily doses (DDD)/1000 inhabitants/day (Klein et al., 2018). The misuse of antimicrobials is common in critical care units among LMICs, including Egypt, which is associated with the emergence of antimicrobial resistance (Elsorady et al., 2022; Global Antimicrobial Resistance and Use Surveillance System (GLASS) Report, 2021). Antibiotics are sometimes sold by community pharmacies without a prescription, which further complicates the problem of antimicrobial resistance (Elsayed et al., 2021; Jamshed et al., 2018; Lambert et al., 2022). The insufficient health literacy levels of the Egyptian population can even make the problem worse (Almaleh et al., 2017; Anwar et al., 2020; Essam et al., 2022; Moustafa & Kassem, 2023). Therefore, applying strict institutional Antimicrobial Stewardship Program is urgently needed to enhance rational antibiotic use (AWaRe Classification, n.d.; Barlam et al., 2016; Cunha, 2018).

Antimicrobial time-out (ATO), previously advocated by The Centers for Disease Control and Prevention (CDC), is a sub-domain of anti-microbial stewardship that involves reviewing antimicrobial therapy after initiation and deciding to continue, adjust, or discontinue according to clinical and microbiologic data (Van Schooneveld et al., 2020). The advantage of ATO is that it can theoretically be implemented without additional resources, personnel, expertise, or outside input, which would be of great value in LMICs such as Egypt and in low-resource settings. However, published data on the efficacy of ATO strategy application is scarce (Richardson et al., 2019; Thom et al., 2019).

Pharmacists’ services have evolved from mere drug dispensing to more individualised and specialised patient care to align with institutional goals and best practices (Al Aqeel et al., 2018; Al Meslamani, 2023a, 2023b; Presley et al., 2019). They follow evidence-based guidelines, formulate local guidelines, design drug use policies, diminish medication errors, champion medication safety initiatives, and assist in treatment decisions (Kassem et al., 2021; Lee et al., 2017; Stasiak et al., 2014; Van der Linden et al., 2020). Their role in reducing medication errors in the vulnerable population of the ICU is expanding (Arredondo et al., 2021; Kharaba et al., 2022).

Clinical pharmacists are potential key players in emergencies and disasters (Erstad, 2021; Li et al., 2021; Perez et al., 2022). Their scope of practice during the pandemic involved providing prevention of infection, providing drug information for healthcare personnel and patients, and optimising the drug therapy (Al Meslamani et al., 2021; Arain et al., 2021; Li et al., 2021; Visacri et al., 2021). In addition, dedicated clinical pharmacists involvement in patient care improves healthcare outcomes and reduces costs (Coralic et al., 2014; Hyland et al., 2020; Morgan et al., 2018; Niznik et al., 2018).

Although most critical care physicians think the advice from a clinical pharmacist is helpful (Shendy et al., 2022), clinical pharmacy services are still not optimally implemented in most of the hospitals in LMICs such as Egypt (Nguyen et al., 2014; Said et al., 2020), especially in low-resource settings (Eldin et al., 2022; Nguyen et al., 2014). Therefore, training clinical pharmacists, especially those working in the ICU may have a positive impact on clinical and economic outcomes (Arredondo et al., 2021; Barlow et al., 2022; Johansen et al., 2016; Nguyen et al., 2014). They can act as an alternative to infectious diseases specialists or infectious diseases clinical pharmacists for antibiotic optimisation when resources are limited (Cunha, 2018; Moody et al., 2012). Moreover, the Society for Infectious Disease Pharmacists emphasises the role of pharmacists in antimicrobial stewardship, even if the pharmacist is not formally trained in infectious diseases (Heil et al., 2016).

The Drug and Therapeutics Committee (DTC) is the highest in the hierarchy of hospital pharmacy management that promotes the best practices in medicine use. It has a wide range of functions, including medication selection for the local formulary, drug information provision to medical staff, drug-use policies development and auditing, and applying standard treatment guidelines. It reduces and manages medication errors (Kabba et al., 2023).

Notably, there is a scarcity of research related to clinical pharmacy interventions or DTC activities to optimise treatment plans and save costs during COVID-19 in LMICs, especially in intensive care units (ICUs). Encouraging multidisciplinary collaboration of pharmacists within the health system by being involved in the DTC was previously encouraged (Arredondo et al., 2021; Cameron et al., 2022; Kabba et al., 2023). They can act proactively in light of the goals of this committee to reduce medication errors and expenditures. However, previously published data usually show the clinical effectiveness of pharmacist-led services provided after DTC implementation, while not analyzing the economic savings of the implementation of these services. Therefore, this study aims, not only to assess the clinical outcomes but also the direct medication cost-savings upon implementing clinical pharmacist-led interventions after the initiation and implementation of the DTC committee during COVID-19 in a low-resource setting in a LMIC. The clinical pharmacist’s interventions aimed to prevent medication errors and to optimise the use of drugs, especially antibiotics in an adult infectious disease ward and an adult ICU of a public COVID-19 isolation hospital.

## 2. Patients and Methods

### 2.1. Study design and setting

#### 2.1.1. Study design

A prospective, comparative pre–post interventional study.

#### 2.1.2. Setting

An infectious disease ward (150 beds) and an intensive care unit (ICU) (50 beds) of a general medium-sized isolation hospital in Egypt.

#### 2.1.3. Duration

From June 2021 to December 2021.

#### 2.1.4. Inclusion criteria

All adults aged 18 years or older, with laboratory-confirmed COVID-19 through polymerase chain reaction (PCR) test of nasopharyngeal swaps and an oxygen saturation of less than 95%, admitted to the isolation ward in the general hospital in Egypt, with an indication of at least one antibiotic, were considered eligible for the Drug and Therapeutics Committee (DTC) interventions.

#### 2.1.5. Exclusion criteria

Ambulatory patients, individuals with human immunodeficiency virus (HIV) infections, pregnant women, those with oxygen saturation of 95% or higher, and those who declined participation were excluded from the study.

### 2.2. Phases of the study

**Figure 1.**
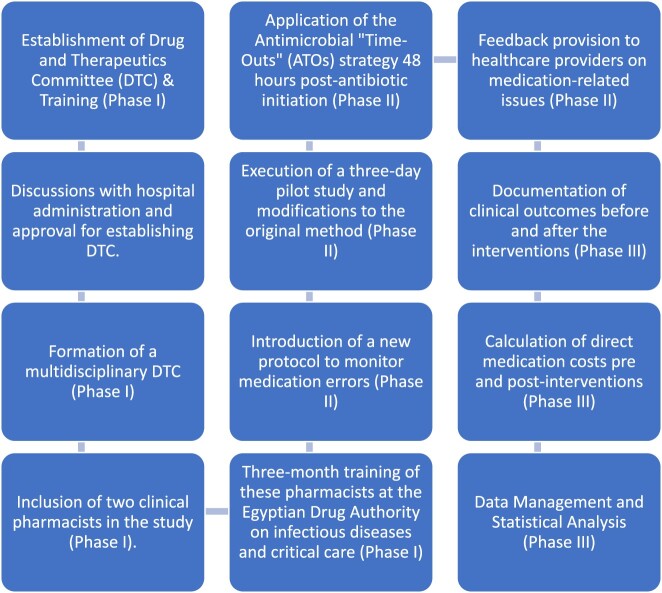
Study procedures and flow.

The study flow is illustrated in Figure 1.

#### 2.2.1. Phase I

Establishment of the DTC and training of its members:

##### 2.2.1.1. Committee members

Multidisciplinary, comprised of physicians, clinical pharmacists, nurses, and other various stakeholders.

##### 2.2.1.2. Training program

Two of the DTC clinical pharmacists received their first formal infectious diseases training and critical care training at the Egyptian Drug Authority for three months. Those two trained clinical pharmacists participated in this study. Infectious diseases training aimed at establishing an enhanced knowledge base regarding antimicrobials. It focused on various aspects of antimicrobial therapy including the stewardship program, selective infections treating algorithms, how to adjust drug doses according to renal and hepatic function, intravenous to oral switching, pharmacokinetic and pharmacodynamic-based dosing, and antibiotic resistance potential. It is worth to mention that there was no infectious disease physician consultant in the selected hospital.

#### 2.2.2. Phase II

DTC-trained clinical pharmacists’ clinical interventions

##### 2.2.2.1. A new protocol establishment

The principal objective of the newly established protocol is to detect and control the rate of medication errors, especially those related to antimicrobial agents.

##### 2.2.2.2. A small-scale pilot study

A pilot study was conducted for three days to evaluate the feasibility and practicality before conducting the final study. As a result of this preliminary study, minor modifications to the original method were made.

##### 2.2.2.3. Interventions

Trained clinical pharmacists in the DTC independently accessed all medical records for clinical information. They reported demographic data (age, gender), care unit (ward or ICU), comorbidities, and pre-intervention prescribed medications. They provided comprehensive and individualised pharmaceutical care to the patients, based on the latest guidelines and protocols. They reviewed the prescribing and dispensing of the medication in 100 medication records against the patients’ clinical data to detect, report, and prevent medication errors. One medication record can contain more than one medication error. Interventions performed by the clinical pharmacist included stopping, starting, or changing the drug dosage. They also aimed at improving the quality of dispensing, as well. They recorded prescribers’ acceptance of the teams’ interventions.

Optimising antimicrobial therapy was done via antimicrobial ‘time-outs' (ATOs) strategy. DTC-trained clinical pharmacists applied the ATOs strategy 48-hours post-antibiotic initiation, after thoroughly assessing the patient characteristics, and reviewing their medical records, in line with a previous study (Van Schooneveld et al., 2020). Details of medication errors were verified by two researchers of clinical pharmacy and pharmacy practice, who reviewed the clinical situations, the medication errors, and the interventions and compared the database with the original data sheets and notes written by the team members.

##### 2.2.2.4. Definitions:

Medication error is defined as ‘any preventable event that may cause or lead to inappropriate medication use or patient harm while the medication is in the control of the healthcare professional, patient, or consumer' (rxr@usp.org, 2014).

A prescription error is defined as any failure in writing the prescription that leads to a wrong instruction (Aronson, 2009).

A dispensing error is defined as the inconsistency between the prescription and the dispensing process (Aronson, 2009; Maharaj et al., 2020).

Prescribing a wrong drug is defined as any irrational, inappropriate prescribing according to guidelines/formulary or prescribing a contraindicated drug (Aronson, 2009; Deawjaroen et al., 2022).

An untreated indication or drug omission means the omission of a potentially beneficial drug that may cause therapeutic inertia (Deawjaroen et al., 2022; Guignard et al., 2015).

Treatment duplication is defined as double prescribing of the same therapeutic group or active ingredient (Deawjaroen et al., 2022).

Low dose is defined as a dose that is lower than the minimal recommended dose for a given indication (Deawjaroen et al., 2022; Guignard et al., 2015).

High dose is defined as a dose higher than the maximal recommended dose for a given indication (Deawjaroen et al., 2022; Guignard et al., 2015).

*Recommendations delivery method:* DTC-trained clinical pharmacists gave feedback to the treating healthcare provider on guidelines-compliant prescribing and medication-related issues via a notification placed in the patient’s file or verbally, referenced by relevant up-to-date guidelines.

#### 2.2.3. Phase III

Effect of pharmacist-led interventions on clinical outcomes and direct medication cost savings to the institution:

##### 2.2.3.1. Clinical outcomes

Patients were followed up from admission to either death or discharge. Also, glucose level, oxygen saturation, platelet count, white Blood cell counts (WBCs), hemoglobin, temperature, respiratory rate, and overall hospital length of stay were documented before and after the intervention.

##### 2.2.3.2. Direct cost saving

Direct medication cost of treating patients admitted during the study period (June–December 2021) was estimated pre- and post-interventions. Cost savings due to interventions were calculated as the cost of therapy pre-intervention minus the sum of the cost of therapy after intervention and the pharmacists’ services cost.

##### 2.2.3.3. Data management

The DTC-trained clinical pharmacists’ team was responsible for collecting data sheets and building the final database. To determine the appropriate sample size for our study, we used the G*Power software, which allowed for a precise estimation based on the expected effect size, power, and significance level. Assuming an effect size of 0.3, a power of 90%, and an alpha level of 0.05, the software recommended a minimum sample size of 88 patients. To account for potential dropouts and ensure robust statistical power, we decided to enroll 100 patients in the study.

#### 2.2.4. Statistical analysis

Our study was designed with specific hypotheses in mind, aiming to evaluate the impact of pharmacist interventions on various outcomes. The primary hypothesis was that these interventions would lead to significant changes in clinical outcomes, cost, and medication errors. To test this, SPSS version 26 was used. For categorical variables, differences were assessed using the Chi-square test or Fisher’s exact test as appropriate. The data were not normally distributed, as indicated by the results of the Shapiro–Wilk test, which showed that the data significantly deviated from a normal distribution (*p* < 0.05). To evaluate changes in median values before and after pharmacist interventions, the Wilcoxon signed-rank test was utilised. This non-parametric test is appropriate for our before-and-after study design, as it compares paired observations and is robust against non-normal distributions of the data.

Furthermore, to visualise differences in median costs before and after interventions, an error bar chart was used. We acknowledged the potential for regression to the mean, a phenomenon where extreme observations tend to move towards the average on subsequent measurements. This effect was considered in our data interpretation, particularly in assessing whether observed changes could be attributed solely to the interventions or partly to this statistical phenomenon. A *p*-value of less than 0.05 was considered statistically significant. However, we recognise that statistical significance does not always equate to clinical significance, and hence, our discussion also considers the practical implications of our findings.

#### 2.2.5. Ethical approval

The study received approval from the Institutional Review Board of Damanhur University (Project No.1023PP69). It was conducted in adherence to the ethical standards of the Declaration of Helsinki (2013). Informed consent was secured from all patients or their substitute legal guardians before including them in the study. The hospital approval was obtained in June 2021 for establishing a DTC for reviewing patients’ files, especially those with prescriptions for antibiotics.

## 3. Results

### 3.1. Demographic data.

During this prospective interventional study, pharmacists monitored 100 patients’ medical records and detected, reported, and prevented medication errors in the infectious disease ward and intensive care unit (ICU) of a general hospital in Egypt through the period from July 2021 to December 2021. The total number of patients included in this study was 100, of which 56 (56.0%) were females, 58 (58.0%) were in the ICU because of complications, such as acute respiratory distress syndrome, shock, or arrhythmia, and 21 (21.0%) had at least 3 comorbidities (Table 1). Additionally, the median age for all patients was 69 years with an IQR of 56.3–75.0 years. Among the patients, 91 (91.0%) had improved clinical outcomes after pharmacists’ interventions. Statistically significant differences in clinical outcomes were observed based on the clinical setting (*p* = 0.009) and the requirement for ventilation (*p* = 0.001). Specifically, among patients treated outside of the ICU (*n* = 42) and those who did not necessitate ventilation (*n* = 52), 100% of them demonstrated improved clinical outcomes (Table 2).

**Table 1.** Basic characteristics of patients (*N* = 100).

Parameter	Total, *n* (%)
Gender	
Female	56 (56.0%)
Male	44 (44.0%)
Age (year), median (IQR)	69 (56.3–75.0)
Weight (Kg), median (IQR)	79.5 (70.0–85.0)
Setting	
ICU	58 (58.0%)
Ward	42 (42.0%)
Need for ventilation	
Yes	48 (48.0%)
No	52 (52.0%)
Comorbidities	
Diabetes	
Yes	54 (54.0%)
No	46 (46.0%)
Hypertension	
Yes	56 (56.0%)
No	44 (44.0%)
Heart failure	
Yes	3 (3.0%)
No	97 (97.0%)
ASCVD	
Yes	8 (8.0%)
No	92 (92.0%)
CKD	
Yes	12 (12.0%)
No	88 (88.0%)
Sepsis	
Yes	1 (1.0%)
No	99 (99.0%)
Number of comorbidities	
0	10 (10.0%)
1	31 (31.0%)
2	33 (33.0%)
3	21 (21.0%)
4	5 (5.0%)

Data are expressed as numbers (n) with percentages (%) unless otherwise stated. ASCVD: Atherosclerotic cardiovascular disease. CKD: chronic kidney disease.

**Table 2.** Distribution of improved and non-improved cases.

Parameter	Improved Clinical outcomes n (%)	Not improved Clinical outcomes n (%)	*P* value
Gender			0.07
Female	48 (85.7%)	8 (14.3%)	
Male	43 (97.7%)	1 (2.3%)	
Age (year), median (IQR)	69 (56.4–74.2)	73 (68.0–75.0)	0.596
Weight (Kg), median (IQR)	79 (70.2–79.6)	80 (70.1 85.0%)	0.823
Setting			**0.009**
ICU	49 (84.5%)	9 (15.5%)	
Ward	42 (100.0%)	0 (0.0%)	
Need for ventilation			**0**.**001**
Yes	39 (81.3%)	9 (18.8%)	
No	52 (100.0%)	0 (0.0%)	
Comorbidities			
Diabetes			0.295
Yes	51 (94.4%)	3 (5.6%)	
No	40 (87.0%)	6 (13.0%)	
Hypertension			0.727
Yes	50 (89.3%)	6 (10.7%)	
No	41 (93.2%)	3 (6.8%)	
Heart failure			0.249
Yes	2 (66.7%)	1 (33.3%)	
No	89 (91.8%)	8 (8.2%)	
ASCVD			0.543
Yes	7 (87.5%)	1 (12.5%)	
No	84 (91.3%)	8 (8.7%)	
CKD			0.595
Yes	12 (100%)	0 (0.0%)	
No	79 (89.8%)	9 (10.2%)	
Sepsis			0.910
Yes	1 (100.0%)	0 (0.0%)	
No	90 (90.9%)	9 (9.1%)	
Number of comorbidities			0.868
0	9 (90.0%)	1 (10.0%)	
1	31 (100.0%)	0 (0.0%)	
2	26 (78.8%)	7 (21.2%)	
3	20 (95.2%)	1 (4.8%)	
4	5 (100.0%)	0 (0.0%)	

Chi-square test or Fisher’s exact test were used as appropriate to calculate *p*-values. Bold *p* value indicates a significant result using chi-square test.

The median hospital LOS for improved cases was significantly shorter at 7 days (IQR 5–10 days) compared to 9 days (IQR 7–12 days) for non-improved cases (*p* = 0.034). Similarly, the ICU LOS median was shorter for the improved group at 3 days (IQR 2–5 days) versus 5 days (IQR 3–7 days) for the non-improved group (*p* = 0.048). Mortality was observed in 2% of improved cases compared to 10% in non-improved cases (*p* = 0.022). Antibiotic consumption was reduced in the improved group, showing a decrease in defined daily doses (DDDs) per 100 bed days from 120 to 85 (*p* = 0.015), indicating more judicious use of antibiotics following pharmacist interventions.

### 3.2. Medication errors reporting

A total of 499 medication orders were documented, of which 238 (47.7%) medication errors were identified, equating to an average of 2.38 errors per patient (Table 3). The most common erroneous antibiotic medications in prescribing included ceftriaxone (n = 68; 30.4%), levofloxacin (*n* = 32; 14.3%), and linezolid (*n* = 23; 10.3%) (as shown in Table 4). Ceftriaxone (*n* = 6; 42.9%) was the most common erroneous antibiotic medication included in dispensing errors of antibiotics. Prescribing errors constituted a significant segment, with 224 (44.9%) errors observed. The most common prescribing errors encompassed wrong drug (*n *= 129, 57.9%), prescribing an excessively high dose (*n* = 67, 29.9%), treatment duplication (*n* = 10, 4.5%), prescribing an insufficient dose (*n* = 10, 4.5%), and overlooking untreated indications (*n* = 8, 3.6%).

**Table 3.** Medication errors identified by pharmacists and pharmacist interventions.

Parameter	Total
Total number of medication orders (A)	499
Total number of medication errors identified (B)	238
Incidence of medication errors (B/A × 100)	47.7%
Medication error per patient	2.38
Number of Prescribing errors	224
Incidence of prescribing errors	44.9%
Number of dispensing errors,	14
Incidence of dispensing errors	2.8%
**Types of prescribing errors**	
Wrong drug	129 (57.9%)
Treatment duplication	10 (4.5%)
Low dose	10 (4.5%)
High dose	67 (29.9%)
Omission (untreated indication)	8 (3.6%)
**Types of dispensing errors**	
Wrong drug	2 (14.3%)
Wrong dose	12 (85.7%)
Total number of pharmacist interventions	315
Categories of pharmacist interventions	
Reducing dose	91 (28.9%)
Increasing dose	6 (1.9%)
Cessation of a medication	141 (44.8%)
Adding a medication	77 (24.4%)

**Table 4.** Types of erroneous medications.

Error type	Erroneous Medication	Total, *n* (%)
Prescribing error		
	Ceftriaxone	68 (30.4%)
	levofloxacin	32 (14.3%)
	linezolid	23 (10.3%)
	azithromycin	19 (8.5%)
	cefepime	18 (8.0%)
	meropenem	16 (7.1%)
	dexamethasone	14 (6.3%)
	prednisolone	14 (6.3%)
	Levofloxacin	12 (5.3%)
	Cefepime	8 (3.6%)
	Other	2 (0.9%)
Dispensing errors		
	Ceftriaxone	6 (42.9%)
	Cefotaxime	2 (14.3%)
	Levofloxacin	2(14.3%)
	Atracurium	2(14.3%)
	Midazolam	2(14.3%)
	Other	1 (7.1%)

Dispensing errors, on the other hand, were comparatively less frequent with 14 identified, accounting for 2.8% of the total. These predominantly pertained to the administration of incorrect doses (*n* = 12, 85.7%), followed by dispensing the wrong drugs (*n* = 2, 14.3%).

### 3.3. Pharmacists’ interventions

A total of 315 pharmacist interventions were recorded. These primarily involved ceasing medications (*n* = 141, 44.8%), dose reductions (*n* = 91, 28.9%), introducing new medications (*n* = 77, 24.4%), and, less frequently, increasing doses (*n* = 6, 1.9%). Examples of clinical scenarios are described in Table 5.

**Table 5.** Clinical scenarios.

Case	Patient profile	Type of medication error	Pharmacist intervention	Outcome
1	Age: 74 Sex: Male Comorbidities: Diabetes, Hypertension, CKD Setting: ICU Medications on Admission: Metformin, Ramipril, Potassium Chloride	Omission: No prescription for antibiotics despite pneumonia. High Dose: Overdosed on potassium chloride.	Recommended the initiation of levofloxacin for pneumonia after assessing the patient’s kidney function. Adjusted potassium chloride dosage based on the patient’s renal function and current potassium level.	The patient’s pneumonia showed signs of improvement with levofloxacin. The corrected potassium dosage prevented potential hyperkalemia. He was shifted to the general ward after 5 days in ICU and was subsequently discharged in stable condition.
2	Age: 52 Sex: Female Comorbidities: Heart Failure Setting: ICU Medications on Admission: Furosemide, Carvedilol	Wrong drug: Administered enoxaparin despite active gastrointestinal bleeding. No Indication: Linezolid was prescribed without the evidence of MRSA or VRSA infection.	Ceased enoxaparin due to the contraindication in the setting of active bleeding. Recommended blood cultures and ensured appropriate antibiotic use. Given the absence of MRSA or VRSA, advised discontinuation of linezolid.	After stopping enoxaparin, there was a cessation of gastrointestinal bleeding. Cultures eventually returned negative for MRSA/VRSA, confirming the appropriateness of stopping linezolid. The patient’s sepsis was managed, and she recovered after a 9-day stay.
3	Age: 78 Sex: Female Comorbidities: Hypertension Setting: Ward Medications on Admission: Ceftriaxone, Linezolid, Paracetamol, Pantoprazole, Dexamethasone	No indication: prescribing linezolid as a first-line antibiotic without indication. Using of linezolid is reserved for MDROs and high-risk patients for MRSA High dose: Ceftriaxone prescribed every 12 hrs. Dose regimen is not appropriate, as ceftriaxone should be taken once daily	Linezolid was discontinued due to absence of indication Dose of ceftriaxone was adjusted to 1 gm / 24 hr.	Sepsis was managed. Cultures returned negative for MRSA/VRSA, confirming the appropriateness of stopping linezolid. WBC was lowered from 16,000 to 5,000 and CRP was lowered from 36 to 0. The patient became not oxygen dependent; oxygen saturation raised from 84% to 96%, and she recovered after a 14-day stay and was discharged.
4	Age: 74 Sex: Female Comorbidities: IHD + AF Setting: Ward Medications on Admission: Linezolid, Cefepime, Enoxaparin, Pantoprazole, Dexamethasone	No indication: Prescribing linezolid as a first line antibiotic without indication as patient on nasal cannula no risk for MRSA High dose: Enoxaparin dose 40 mg/hr (Not appropriate dose for AF)	Discontinue linezolid. Using of linezolid is reserved for MDROs and MRSA risk and to be at last line defense Adjust enoxaparin to be 60 mg / 12 hr as dose is not appropriate according to the indication of AF	Cultures returned negative for MRSA/VRSA, confirming the appropriateness of stopping linezolid. Patient became room air dependent and no more oxygen supply as his oxygen saturation increased from 85 to 95 and she was discharged after 6 days stay
5	Age: 75 Sex: male Comorbidities: DM + stroke and high ammonia level + right foot edema Setting: Ward Medications on Admission: Ceftriaxone, Fondaparinux, Dexamethasone, Pantoprazole, Remedesvir, Atorvastatin, Acetyl salicylic acid, Captopril, Bisoprolol, Baricitinib	Omission: no prescription for lactulose, gastrobiotic or lactulose enema despite high ammonia level	Lactulose, gastrobiotic and lactulose enema were added to his regimen	Patient level of consciousness was raised and he became more alert and was discharged after 6 days stay

### 3.4. Pre-intervention versus post-intervention parameters

Following the intervention, several significant changes were observed in the evaluated parameters. The number of prescribed medications pre- and post-intervention remained relatively consistent: 5 (IQR: 4–6) compared to 5 (IQR: 3.25–6); this difference was not statistically significant (*p* = 0.157).

Glucose levels displayed a notable reduction from a median of 407.5 (IQR: 90.7–548.7) to 130 (IQR: 110–179), though this change was not statistically significant (*p* = 0.109) (Table 6).

**Table 6.** The impact of pharmacist interventions on the number of prescribed medication and clinical parameters.

Parameter	Pre-intervention, median (IQR)	Post-intervention, median (IQR)	*P* valu
Number of prescribed medications	5 (4–6)	5 (3.25–6)	0.157
Glucose level	407.5 (90.7–548.7)	130 (110–169)	0.109
Oxygen saturation	88 (83.5–89.5)	96 (94.5–96.5)	**0.011**
Platelet count	79000 (70,000–86,200)	115,000 (110,000–115,000)	0.180
Temperature, ^o^C	39.5 (39–39.8)	35.0 (30.0–36.0)	**0**.**001**
Respiratory rate	35 (30–36)	22 (22–22)	**0**.**002**
WBCs	19,800 (18,000–24,000)	8600 (7200–9700)	**0**.**001**
Haemoglobin	7.1 (7.1–7.1)	9 (9.0–9.0)	0.317

Wilcoxon signed-rank test was used to calculate *p* values. Bold *p* values indicate significant results. IQR: interquartile range.

Oxygen saturation exhibited a significant increase from 88% (IQR: 83.5–89.5) pre-intervention to 96% (IQR: 94.5–96.5) post-intervention (*p* = 0.011). While the platelet count rose from 79,000 (IQR: 70,000–86,200) to 115,000 (IQR: 110,000–115,000), this change was not statistically significant (*p* = 0.180). Temperature demonstrated a marked reduction post-intervention, shifting from 39.5°C (IQR: 39–39.8) to 35.0°C (IQR: 30.0–36.0) (*p* = 0.001). The respiratory rate also saw a significant decrease, moving from 35 (IQR: 30–36) to 22 (IQR: 22–22) post-intervention (*p* = 0.002). White blood cell (WBC) counts declined substantially from 19800 (IQR: 18,000–24,000) pre-intervention to 8600 (IQR: 7200–9700) post-intervention. However, changes in hemoglobin levels did not achieve statistical significance (*p* = 0.317).

**Figure 2.**
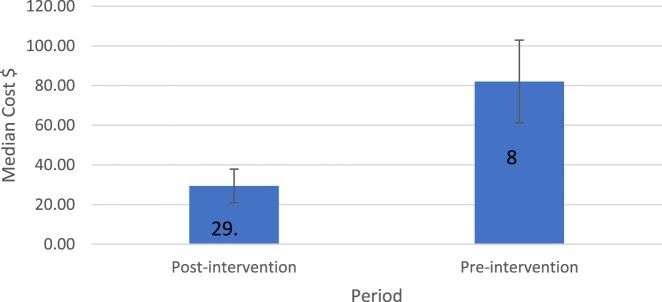
Median of cost ($) by pharmacist intervention (pre vs post).

Antibiotic expenditure was retrieved from the hospital database. Without the intervention, the medication cost was supposed to be 12695.9 dollars. The medication cost after the intervention was 6195 dollars and the cost for pharmacist service in the intervention was 2483 dollars. The wage per work hour of a pharmacist was 2.6 dollars. The total length of stay of all patients was 955 days and each of the two clinical pharmacists worked for 30 minutes per case each day. Therefore, the total working hours dedicated to the service by the two clinical pharmacists were 955 hours, taking into account that the case workup will take approximately 30 minutes (including medication review, feedback to the physician, mind storming with the other clinical pharmacist, and documentation). About 4017.8 dollars were saved after subtracting the cost of pharmacists’ services. The findings showed a statistically significant decrease in the median cost of treatment after pharmacist interventions (82.0$ pre-intervention vs. 29.3$ post-intervention) (Figure 2).

## 4. Discussion

Developing countries are in greater need of urgent healthcare plans due to their considerable health disparities (Al Meslamani et al., 2021; Gwatkin, 2017). Although clinical pharmacists can have a powerful impact on the optimisation of therapeutic regimens and saving medication costs, clinical pharmacy services in low-resource settings in developing countries are not actively implemented (Bronkhorst et al., 2020; Shrestha et al., 2019). Therefore, this study aimed to fill the gap of knowledge regarding the impact of clinical pharmacists’ interventions as members of a Drug and Therapeutics Committee (DTC) on improving clinical outcomes and reducing medication costs in an isolation governmental hospital during COVID-19, in a low-to-middle income country (LMIC).

In the current study, the DTC-trained clinical pharmacist interventions significantly improved patients’ clinical outcomes and reduced direct medication costs. Improved patients’ clinical outcomes reported included oxygen saturation, respiratory rate, fever, and WBC count in 91% of patients. Statistically significant differences in clinical outcomes were observed among patients treated outside of the intensive care unit (ICU) and those who did not require mechanical ventilation (100% of them demonstrated improved clinical outcomes). This may be attributed to the fact that ICU patients are usually more vulnerable than patients in the medical ward setting (Santhosh et al., 2019) and that the use of ventilator-associated events may be associated with worse clinical outcomes (Weinberger et al., 2021). It was reported previously that clinical pharmacist involvement in the ICU led to a significantly lower length of stay, medication costs, patient care costs, and risk of mortality (Khalili et al., 2013; Leguelinel-Blache et al., 2018; MacLaren & Bond, 2009).

There was no significant statistical difference between the number of prescribed medications pre- and post-intervention, in line with a previous study (Liou et al., 2021). Expenditures were measured by comparing medication costs before and after the implementation of the intervention, in line with a previous study (Beardsley et al., 2012). Calculations were based on prescriptions and administrations instead of purchasing data, which was previously recommended (Barlam et al., 2016).

The adopted antimicrobial ‘time-outs' (ATOs) strategy, a sub-domain of the anti-microbial stewardship program, was feasible and accepted. Stewardship strategies can save antibiotic medication costs (Malani et al., 2013; Nowak et al., 2012), in line with our study. However, some previous studies failed to demonstrate a significant healthcare resources reduction (Al-Hashar et al., 2018; Cypes et al., 2021; Liou et al., 2021). Even with fewer resources and supports and no dedicated pharmacist time, our DTC-trained clinical pharmacists-led antimicrobial stewardship interventions have produced significant reductions in antimicrobial use and expenditures, in line with a previous study with limited resources (Malani et al., 2013). There may have been a misconception that limited resources settings are incapable of applying antimicrobial stewardship programs (Lim et al., 2014). The role of pharmacists in the optimisation of antimicrobial use in ICU should be emphasised in developing countries (Shendy et al., 2022), even if the pharmacist is not formally trained in infectious diseases (Heil et al., 2016).

DTC-trained clinical pharmacists reported prescribing errors constituted 44.9% of all reported errors. Most of the medication errors that occurred in critically ill patients happened during prescribing (Merino et al., 2013). Non-antimicrobial medications involved in medication errors included remdesivir, insulin, and anticoagulants (heparin enoxaparin, and fondaparinux) which were among the top medications implicated in errors or reported to need careful prescribing in previous studies (Patil et al., 2022; USP Drug Safety Review: Top 10 Drugs Involved in Medication Errors, 2023; Wittich et al., 2014). In addition, most patients with COVID-19 treated in ICU received antiviral therapy (10). Remdesivir use in patients with an estimated glomerular filtration rate of less than 30 mL per minute may increase the potential for hepatic and renal toxicity (Bhimraj Adarsh & Falck-Yetter, 2023). Its use is not recommended in severe liver and kidney diseases (Kale et al., 2023).

The most common prescribing errors encompassed the wrong medication. DTC-trained clinical pharmacists stopped these inappropriate medications, in line with a previous study (Martin et al., 2018). In a previous study conducted in a nephrology ward in Iran, the majority of the clinical pharmacists’ interventions aimed to correct a wrong medication (Vessal, 2010). Overlooking untreated indications also was reported, in line with a previous study (Klopotowska et al., 2010).

The prescriptions were written manually, which might contribute to a higher incidence of medication errors versus using electronic prescribing (Riaz et al., 2014; Volpe et al., 2016). Medication errors in the ICU were higher, in line with a previous study (Suclupe et al., 2020). Most of the patients in this work were elderly. Moreover, we found statistically significant differences in clinical outcomes among patients treated outside of the ICU versus those treated inside the ICU. Patients who require ICU care are more likely to be older and have underlying comorbidities, including hypertension, diabetes, cardiovascular disease, and cerebrovascular disease, compared with non-ICU patients (Tang & Wang, 2020; Wang et al., 2020); which makes them more vulnerable to medication errors (Fick & Semla, 2012).

The frequent empirical prescribing of broad-spectrum antibiotics in the ICU settings in Egypt contributes to antimicrobial resistance as reported by a previous study in which nearly half of critical care patients received ≥3 antibiotics (Elsorady et al., 2022). The hospital in which this study was performed represents a setting of increased need for stewardship because small, nonteaching hospitals have a high rate of antibiotic use (Baggs et al., 2016). Most patients with COVID-19 were treated with empirical broad-spectrum antibiotics because the laboratory diagnosis of COVID-19 takes time, and it could be difficult to distinguish the disease from other bacterial and viral pneumonia (Guan et al., 2020; Huang et al., 2020; Wang et al., 2020).

Antibiotics with the highest number of pharmacist-led interventions included linezolid, ceftriaxone, cefepime, and levofloxacin. Linezolid, levofloxacin, and ceftriaxone were among the most prescribed antibiotics in Egypt during the pandemic (Elsayed et al., 2021). The World Health Organization (WHO) listed linezolid as a reserve antibiotic (used as a last resort) (AWaRe Classification, n.d.). It should be reserved for targeting multi-drug resistant gram-positive bacteria, including MRSA, and should not be used as the first line in the absence of contraindication to vancomycin (Elsorady et al., 2022; Matrat et al., 2020). It was among the medications that pharmacists intervened upon during a previous study (Al Meslamani et al., 2021). Clinical pharmacists recommended stopping linezolid because of the absence of evidence of Methicillin-resistant Staphylococcus aureus (MRSA) or Vancomycin-resistant Staphylococcus aureus (VRSA) infection, which was confirmed subsequently by cultures (Table 5; cases 2,3, and 4). Prolonged linezolid therapy is associated with resistance and it is recommended to use high-dose, low-resistance potential antibiotics for the shortest duration to achieve clinical elimination of the infection (Cunha & Opal, 2018).

Ceftriaxone, cefepime, and levofloxacin were listed by WHO as a watch (as they have a high potential to develop antimicrobial resistance) antibiotics (AWaRe Classification, n.d.). This finding correlates with previous studies performed in Egyptian critical care units revealing that cephalosporins were among the most commonly consumed antibiotics (Elsorady et al., 2022; Shawki et al., 2022). In a previous study performed during COVID-19 in Egypt, some of the surveyed community pharmacists reported dispensing a non-appropriate dose of ceftriaxone (Elsayed et al., 2021). Levofloxacin and ceftriaxone wide and irrational use before and during the pandemic in both the inpatient and the outpatient settings could have contributed to the increased antimicrobial resistance (King et al., 2019; Medic et al., 2023). Physicians accepted all of the DTC-trained clinical pharmacists-led recommendations, in line with a previous study (Malani et al., 2013).

Clinical pharmacists’ interventions in the current study included cessation of medications. Deprescribing is a complex decision (Balsom et al., 2020; Martin et al., 2018), that is rarely discouraged by healthcare professionals (Martin et al., 2018; Tannenbaum et al., 2014). Physicians may have poor knowledge of medications’ pharmacodynamics and pharmacokinetic properties and poor adherence to the latest therapeutic guidelines (Beshyah et al., 2017; Solà et al., 2014). Pharmacists increased prescribers’ perceptions of best practices which increases doctors’ motivation, enables them to increase the standard of their prescribing practice, and improves their capabilities (McLellan et al., 2016). The clinical pharmacists’ discussions with physicians can significantly reduce medication error occurrence (Shaker et al., 2023).

We recommend that the government and policymakers in LMICs should help healthcare providers optimise the healthcare provision and promote rational drug use by enabling the establishment of DTC due to its vital role in this aspect, especially during disasters and pandemics (Alefan et al., 2019; Moustafa & Kassem, 2023).

## 5. Conclusions

Drug and Therapeutics Committee (DTC) implementation resulted in significant improvement in clinical outcomes and direct medication cost savings via reducing irrational medication use, especially in antibiotic use which can decrease antibiotic resistance. DTC-trained clinical pharmacists’ team successfully ensured the safe dispensing and the optimum prescribing of medications to patients which resulted in an improvement of the vital signs and laboratory findings of the critical care patients, in addition to reducing the unnecessary medication costs. This study adds new value to clinical pharmacists’ proactive capabilities in providing high-quality care during disasters in low-resource settings in developing countries. This DTC service is of great importance to patients in critical care who are at greater risk of medication errors and antibiotic misuse. Implementing targeted education in specific clinical areas, such as infectious diseases should be promoted to enhance pharmacists’ clinical skills for achieving better patient care, especially during disasters.

## 6. Limitations

This study has several limitations. First, the study hospital was a single-center general hospital in a developing country during the COVID-19 pandemic in Behera, Egypt; therefore, the result may have limited generalizability due to its unique set of resources and geographic setting. Second, this study is a prospective interventional pre–post study. Therefore, there is a need for randomised controlled trials to confirm our findings. The main strength of this work is that we assessed two stages of medication use, prescribing, and dispensing, whereas previous research papers have focused mostly on one stage only.
